# Sponge city construction and population health

**DOI:** 10.3389/fpubh.2024.1285568

**Published:** 2024-01-29

**Authors:** Yue Li

**Affiliations:** School of Public Administration and Policy, Renmin University of China, Beijing, China

**Keywords:** sponge city construction, population health, therapeutic effect, quasi-natural experiment, difference-in-differences model, China

## Abstract

**Introduction:**

This paper focuses on the construction of sponge cities and their effects on population health. Sponge cities in China both solve stormwater problems and are a systemic transformation in the urban construction paradigm, addressing related issues arising from high-speed urbanization. Whether sponge city construction in China can promote population health has received scant attention. Most previous studies have focused on urban environments and population health, with few exploring the potential effects on population health caused by urban environment changes due to urban policies. This study hypothesizes that sponge city construction improves the urban environment, and thus, population health.

**Methods:**

Using panel data from 119 prefecture-level cities between 2011 and 2019 and the China Labor-force Dynamics Survey (CLDS), based on China’s sponge city pilot policy, a quasi-natural experiment is conducted using Difference-in-Differences (DID) model to identify the health effects of the sponge city policy.

**Results:**

The findings show that sponge city pilot policy not only reduced ecological environment pollution and promoted the quality of built environment, but also significantly improved population health by 10.4%. This mechanism is mainly due to the restorative effects of the built environment.

**Discussion:**

The health effects vary across city administrative levels, and especially among non-older adults and local populations. Compared with the cities at higher administrative level, the health effect in lower administrative level is significantly positive, indicating that there is a diminishing marginal effect of sponge city construction. This study extends the causal identification chain of the impact of urban environment on population health to urban policies and provide insights into policy objectives for sponge city construction.

## 1 Introduction

Globally, urban stormwater concepts such as low-impact development (LID) and best management practices (BMPs) were proposed in developed and developing countries in response to urban sustainability and ecological environmental issues (1, 2). In China, the sponge city concept both aims to solve stormwater problems and are a systemic transformation in the urban construction paradigm, addressing related issues arising from high-speed urbanization (3). Metaphorically, a sponge city soaks up stormwater，or specifically, a city with good “elasticity” in adapting to environmental changes and coping with natural disasters. Thus, it combines LID, BMPs, and other Western stormwater management ideas with the philosophical wisdom of ancient Chinese practices. The “sponge” includes water systems such as rivers, lakes, pools, green belts, and parks. Their essence lies in altering the traditional urban construction pattern amid rapid urbanization, ultimately achieving sustainable city development and healthy urbanization (3).

Understanding the relationship between urban environments and population health has always been a classic topic in the fields of health geography and urban planning (4–6). The issues of urban environment characteristics may affect population health through a multiplicity of processes including influencing health-related behaviors through features such as walkability, the location of parks and green spaces, are widely recognized and well documented (7–9). Overall, current studies on the relationship between urban environments and population health primarily follows the logic of ecological and built environment respectively, neglecting the impact on population health of the environmental change caused by urban policies. Fundamentally, urban construction concepts and other spatial policies are the primary factors shaping the urban environment (10–12).

Unlike urban stormwater management in most western countries, sponge cities in China both solve stormwater problems and are a systemic transformation in the urban construction paradigm, which is aims to create healthier and more sustainable urban environments for individuals. However, existing research on sponge city issues primarily focuses on strategies and application fields (13, 14), whether and why the sponge cities pilot policy affects the urban environment—and, thus, population health—still received scant attention.

To answer this question, we conduct a quasi-natural experiment based on the sponge cities pilot policy in China. To regulate and promote the healthy development of sponge cities, the “Ministry of Housing and Urban–Rural Development” has launched a national sponge city pilot project. To date, the Chinese government issued four batches of national sponge cities in 2015, 2016,2021 and 2022 respectively, providing policy basis for the construction of sponge cities in China. After the pilot cities were set up, China’s sponge city construction is currently well underway. In a word, this policy is not only crucial to promoting the construction of sponge cities in China, as the transformation of urban construction concepts, it also has great research value, providing a typical scenario for this study to explore how urban policies influence the urban environment and subsequently affect population health.

The main research process of this paper is as follows. First, our study analyzes the direct effect of construction of sponge cities on urban environment and the reliability of the basic conclusion is verified by a series of robustness tests. Second, from an individual perspective and considering the diverse characteristics of cities and individuals, we analyze the health effects of sponge city construction, conduct a heterogeneity analysis, and explore the diversified effects and the prerequisites for these effects. Third, we discuss and verify two important mechanisms, attempting to explain why sponge cities can impact individual health.

Compared to existing literature, the contributions of this paper are as follows: First, in this paper, the causal identification chain of the impact of urban environment on health is extended to urban policy, and the impact of the dual changes of ecological environment and built environment caused by sponge city construction on population health is identified. Our study enriches the empirical literature on public policy, particularly urban policy, and its impact on population health.

Second, using panel data from 119 prefecture-level cities in China from 2011 to 2019 and nationally representative data from the China Labor-force Dynamics Survey (CLDS), this paper juxtapose the level of city and individual, examining the policy effects of sponge city on urban environment and individual health, respectively. It offers insights for creating sustainable urban environments and healthier populations through sponge city initiatives.

Third, our study is the first to focus on the health effect of sponge city construction. As a new and important topic in the sustainable and healthy development, there is no existing literature on this topic. Therefore, this study first presents and analyzes it using systematic quantitative research methods to fill the gap in the sponge city research area.

The remainder of this paper is organized as follows. Section 2 is the literature review, outlines of research framework and hypothesis. Section 3 is the research design. Section 4 is the empirical analysis, including benchmark regression results, robustness test and mechanism analysis; Section 5 is heterogeneity analysis; Section 6 is the discussion. Section 7 is conclusion and policy implications.

## 2 Literature review and theoretical analysis

### 2.1 Literature review

Urban environment has always been linked to population health (6). As the most fundamental and dynamic productive resource, population health involves the sustainable pursuit of human social development and well-being. Over the past few decades, a significant body of literature has emerged on the relationship between urbanization and physical and mental health (15). However, urbanization has been proved to have dual impacts on individual health, with positive aspects such as superior amenities and opportunities, as well as negative aspects such as a disconnect from nature (16), exposure to ecological pollution, and unhealthy lifestyles. As the most direct and profound factor, the urban environment and its detrimental effects on population health—including poor air and water quality, physical exposure, and chronic stress—are widely recognized and well-documented (17, 18). These factors can directly or indirectly affect population health during the continuous urbanization process.

Overall, researchers have extensively examined how the urban environment affects population health, primarily following the logic of the ecological or built environment (19–21). Studies on the ecological environment have mainly focus on pollution, including air pollution, and the effects of natural disasters caused by climate change. These studies often highlight the negative health effects of environmental pollution, such as reduced bodily function, increased risk of respiratory diseases such as lung cancer, higher health care costs, and a declining quality of life for residents (22–24). Research on the impact of the built environments on individual health primarily stems from urban planning and geography. Among them, green space as an important part of the urban built environment, and a large number of studies have shown that urban green space promotes the residents’ health by enhancing physical health perception and reducing the incidence of respiratory diseases (25).

From the perspective of governments and public policy scholars, population health issues and their associated costs are a pressing concern in the worldwide (23). Among the various factors influencing population health, public policy can be defined as the result of a governmental process that occurs in response to events over time or at a specific point in time. In turn, it may affect the physical or social environment, thereby influencing health outcomes in neighborhoods, cities, and metropolitan regions (26). Growing evidence suggests that ultimately, social and economic policies may affect health. Most previous studies have primarily concentrated on the impacts of social policies that may indirectly affect health by impinging on social or economic outcomes (27, 28). Indeed, when policies directly target the urban environment, their impact on population’ health is likely to be direct. Overall, from the perspective of public policy, a significant question in the current urban development process is what kind of urban environment is most conducive to population health. Recently, Scholars in public policy and administration have conducted explorations into the health effects of various urban policies such as low-carbon city (29), smart city (30), healthy city (31, 32), even sanitary city (33). While most of the aforementioned literature focuses only on the individual level, overlooking the urban environmental changes caused by policies.

### 2.2 Theoretical analysis

Sponge city construction in China adheres to the idea of low-impact development and aims to achieve the sustainable development of urbanization. In terms of urban environments, improving gray infrastructure, such as drainage networks and rainwater pumping stations, not only beneficial for eliminating sudden risks that affect public health, such as the widespread waterlogging caused by various natural disasters, including heavy rain and floods, but also helps reduce the pollution of ecological environment. Meanwhile, the construction of green infrastructure, such as transforming street green spaces into parks and increasing urban greenways, can also enhance the build environment’s quality, positively affecting health. Overall, theoretically, China’s Sponge City pilot policy can improve population health by mitigating the negative feedback of ecological pollution and increasing the positive impact of the built environment. Based on the above analysis, we propose the following hypothesis:

*H1:* Sponge city construction promotes the urban environment, and thus, population health.

**Figure 1:**
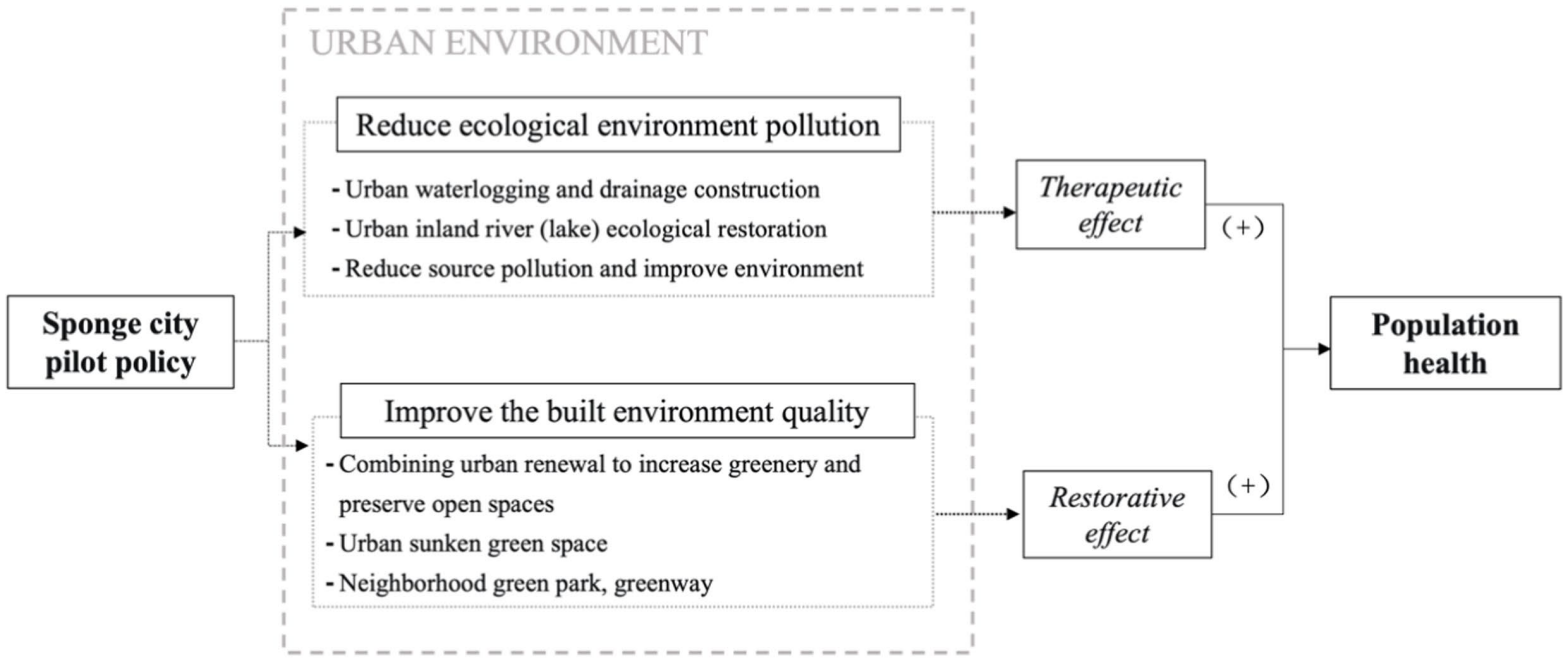
Mechanisms of sponge city construction on population health.

As a new concept and form of evolution and development for traditional cities, sponge cities can potentially and effectively enhance population health through the ecological environments’ therapeutic effects and built environments’ restorative effects. This study proposes that the mechanism through which sponge city pilot policies impact individual health may follow the linear logic of “*sponge city construction–the urban environment–two major effects–population health*” (Figure 1).

#### 2.2.1 Therapeutic effect

Evidence increasingly suggests that exposure to nature has beneficial effects on population health and well-being (34, 35). In both eastern and western cultures, rapid urbanization landscapes devoid of natural elements are often representative of negative health effects (36). Empirical studies have indicated that people are happier in natural than building-dominated environments (37). Only by returning to nature can people obtain fresh air, clean water, and plant resources, and achieve the ideal state of “harmony between heaven and humans” (38). The ecological environment’s critical role in promoting population health has gained consensus in academia, with recognition of its significant impact as a determinant of health (39). The ecological environment is also crucial in promoting positive emotions, reducing stress, and improving population health and well-being (40), with proven therapeutic effects on population health (16). Landscapes in the ecological environment can also be considered therapeutic (41), with green plants and bodies of water as key elements. Based on this, we propose the following hypothesis:

*H1a:* Sponge city construction can enhance population health through its therapeutic effects.

#### 2.2.2 Restorative effect

Rapid urbanization has led to high-density development that not only pursues parallel functions and efficiency to expand urban areas, but also continuously squeezes and encroaches on natural spaces. This contributes to sedentary lifestyles and exacerbates health problems (42). Green spaces have been recognized as an integral part of the built environment, existing between semi-natural and natural areas. These have been associated with numerous health benefits, as they contribute to restoring the environment, promoting biodiversity (43), and having a calming effect (44). In recent years, urban green spaces used to experience nature have declined in both quality and quantity, although literature considers them an ideal place for relieving stress and restoring focus (16). Numerous studies have demonstrated that urban green spaces, and particularly public spaces, are vital in built environments. They effectively reduce incidences of cardiovascular and respiratory diseases (45), encourage physical activity (46), alleviate stress (47), improve overall health (48), and promote positive, healthy lifestyles (49). Therefore, we propose the following hypothesis:

*H1b:* Sponge city construction can enhance population health through its restorative effects.

Furthermore, China’s regions significantly vary in terms of their economic levels and resource endowments, which are intertwined with their administration and urbanization level (50). Cities with higher administration and urbanization level often have more advanced health care facilities, resulting in diminished marginal health effects from the same policies. Hence, this hypothesis suggests that the health effect from sponge cities’ construction may decrease as the administrative level increases. Based on the law of diminishing marginal effects, we propose the following hypotheses:

*H2:* Less administrative cities significantly improve their population health.

*H3:* Less urbanized cities significantly improve their population health.

## 3 Research design

### 3.1 Model specification

According to policy documents “Guiding Opinions on Promoting the Construction of Sponge Cities” and the “Notice on Carrying Out the Pilot Work of Sponge City Construction Supported by the Central Finance “, since 2015, four batches of sponge city pilots have been successively launched by the Ministry of Finance, Ministry of Housing and Urban–Rural Development, and Ministry of Water Resources in 2015, 2016, 2021 and 2022, respectively. Concerning the implementation period, we select the first and second batches of sponge city pilot as the sample for the treatment group. Since the interval between April 2015 and April 2016 is relatively short, there has been no significant change or difference in China’s socio-economic development stage and development tasks during this period. We set 2015 as the starting point of sponge city construction pilots. If the sponge city pilots achieve the goals, it indicates that the policy may have a certain enhancing effect on the ecological and built environments of the pilot cities, which could potentially impact the population health of these cities.

Our study focuses on whether and how the construction of sponge cities affects urban environment and population health, simply comparing the urban environment and population health before and after the pilot may produce a bias because some other factors can affect the urban environment and individual health during the same period. To cope with this problem, this paper adopts the method of quasi-natural experiment for reference to construct a difference-in-differences (DID) model, which has been widely used in recent years for analyzing policy effects. The core idea is to treat the implementation of sponge city pilot policy as a “quasi-natural experiment” that is external to the urban system. The samples affected by the policy were the treatment group (Table 1), otherwise the control group (Table 2).

**Table 1:** Sponge city pilot list.

Batch	Sponge city pilot
First batch (16 cities) (April 2015)	Qian’an, Baicheng, Zhenjiang, Jiaxing, Chizhou, Xiamen, Pingxiang, Jinan, Hebi, Wuhan, Changde, Nanning, Chongqing, Suining, Guian New Area, Xixian New Area
Second Batch (14 cities) (April 2016)	Beijing, Tianjin, Dalian, Shanghai, Ningbo, Fuzhou, Qingdao, Zhuhai, Shenzhen, Sanya, Yuxi, Qingyang, Xining, Guyuan

Guian New Area and Xixian new area are not prefecture-level cities and were excluded from the treatment group sample.

**Table 2:** Sponge city treatment and control panel data.

	Treatment group (pilot city)	Control group (non-pilot city)
$T_{pre}$ (Before Treatment)	${\bar{y}}_{1,pre}$	${\bar{y}}_{0,pre}$
$T_{\mathrm{post}}$ (After Treatment)	${\bar{y}}_{1,\mathrm{post}}$	${\bar{y}}_{0,\mathrm{post}}$

The implementation of China’s sponge city pilot policy, on the one hand, create difference in the urban environment and population health within the same pilot city before and after its implementation, that is $\left({\bar{y}}_{1,\mathrm{post}}-{\bar{y}}_{1,pre}\right)$ ; On the other hand, it may also create differences in these two indicators between pilot and non-pilot cities at the same time point, that is $\left({\bar{y}}_{1,\mathrm{post}}-{\bar{y}}_{0,\mathrm{post}}\right)$ . Regression estimates based on DID model can effectively control the influence of other simultaneous policies and pre-existing differences between pilot and non-pilot cities, thereby identifying the net impact of policy shocks on the urban environment and population health (ATT).

$$\begin{array}{l} ATT=\left({\bar{y}}_{1,\mathrm{post}}-{\bar{y}}_{1,pre}\right)-\left({\bar{y}}_{0,\mathrm{post}}-{\bar{y}}_{0,pre}\right)\\ \;=\left({\bar{y}}_{1,pre}-{\bar{y}}_{0,pre}\right)-\left({\bar{y}}_{1,\mathrm{post}}-{\bar{y}}_{0,\mathrm{post}}\right) \end{array}$$

#### 3.1.1 Urban environmental effect model

Based on the city level, this paper first investigates the impact of sponge city construction on the urban environment. The econometric model is constructed as follows:

(1) $$CE_{ct}=\alpha_{0}+\alpha_{1}SC_{ct}+\alpha Z_{ct}+city_{c}+year_{t}+\varepsilon_{ct}\text{.}$$

Among them, *c* is the city code ranging from 1 to 119 and *t* is the year code ranging from 2011 to 2019. *CE_ct_* (City Environment) is the dependent variable, *SC_ct_* (Sponge City) is the independent variable, and *Z_ct_* are a group of control variables, *InGDP_ct_* (economic development level), *IL_ct_* (industrialization level), *UL_ct_* (urbanization level), CS*_ct_* (city size), and *PD_ct_* (population density). *α_0_* is the constant term. *α_1_* are estimated coefficients. *city_c_* and *year_t_* indicate the city and year fixed effects. *ε_ct_* is the error term (Table 2).

#### 3.1.2 Individual health effect model

Based on the model (1), our study further investigating sponge city construction’s influence on health at the individual level, with our model defined as follows:

(2) IHit=β0+β1ISCit+βXit+φi+yeart+εit.

Among them, *i* is the individual code ranging from 1 to 8,131 and *t* is the year code ranging from 2011 to 2019. *IH_it_* (Individual Health) is the independent variable, *ISC_it_* (Sponge City where Individual located) is the independent variable, and *X_it_* are a group of control variables, *IUL_it_* (Urbanization level where Individual located), *IUZ_it_* (Urban size where Individual located), *IW _it_* (Industrial Wastewater where Individual located), *IP*GDP *_it_* (*Per capita* GDP where Individual located), *IPD_it_* (Population Density where Individual located) and *IHB_it_* (Hospital Beds per 10,000 people where Individual located). Furthermore, *AGE _it_* (Age), *GEN_it_* (Gender), *MAR_it_* (Marriage), *HS_it_* (Hukou Status) and *EDU_it_* (education) were selected as individual characteristic control variables. *β_0_* is the constant term. *β_1_* are estimated coefficients. *φ_i_* and *year_t_* indicate the individual and year fixed effects. *ε_it_* is the error term.

### 3.2 Variables and data

In this study, in Equation 1, this paper use the city’s park or green spaces and average annual concentration of *PM2.5* to measure the built environment’s quality and ecological environmental pollution indicators, respectively (50, 51); Construct dummy variable according to sponge city pilot as independent variable in main research. The China Urban Statistical Yearbook and EPS database (2011–2019) were the main data sources used to examine sponge city pilot policy effect at the prefectural level.

For dependent variable in Equation 2, this paper used self-rated health to represent individual health level, and construct dummy variable according to individual whether stay at sponge city as independent variable. Moreover, to analyze and verify the influence mechanism, two mediating variables - that is - residents’ wellbeing and outdoor exercise duration are chosen. The control variables are the same in Equation 2. The data is from the China Labor Dynamics Survey (CLDS) conducted by the Sun Yat-sen University Social Survey Center every 2 years, which is a nationally representative individual questionnaire. For example，the variable self-rated health come from the following question: “How do you perceive your current health status?” Responses ranged from one (“very healthy”) to five (“very unhealthy”). This assignment method was applied consistently to CLDS data from 2012, 2014, 2016, and 2018. Individual-level control variables and mediating variables were all obtained from the CLDS database using the same methods. At last, we matched the CLDS data to prefectural level city data. A panel dataset of 8,131 observations was obtained by considering the data’s consistency, availability, and operationalization. Table 3 summarizes the statistics for these parameters.

**Table 3:** Definition and summary statistics of variables.

Variable	Definition	*N*	Mean	S.D.
Prefectural level				
*SC*	Sponge City (yes = 1)	1,071	0.121	0.327
*PARK*	Park or green area (in 10,000 hectares)	1,071	0.235	0.418
*InPM2.5*	*PM* 2.5 (μg/m^3^)	1,071	3.826	0.375
*InGDP*	GDP (in 100 million yuan)	1,071	10.772	0.558
*IL*	Secondary Industry/GDP	1,071	0.455	0.105
*UL*	Urbanization Level	1,071	0.563	0.152
*CS*	City Size (in 10,000 people)	1,071	5.946	0.743
*PD*	Population Density	1,071	8.039	0.646
Individual level				
*IH*	Individual self-rated Health	8,131	3.741	0.89
*RW*	Residents’ Wellbeing	6,678	3.687	0.58
*OE*	Outdoor Exercise Duration (in 1 h)	6,688	0.887	0.54
*ISC*	Sponge City (yes = 1)	8,131	0.121	0.326
*IUL*	Urbanization Level	8,131	0.679	0.168
*IUZ*	Urban size (in 10,000 people)	8,131	6.173	0.542
*IW*	Industrial waste water emissions (in 10,000 tons)	8,131	8.953	0.938
*IPGDP*	*Per capita* GDP (in 10,000 yuan)	8,131	11.296	0.5
*IPD*	Population density	8,131	8.254	0.687
*IHB*	Number of hospital beds per 10,000 people	8,131	10.217	0.674
*AGE*	Year	8,131	42.193	13.724
*GEN*	male = 1, female = 2	8,131	1.543	0.498
*MAR*	Marital status (married = 1)	8,131	0.372	0.483
*HS*	Household registration type (local = 1)	8,131	0.681	0.466
*EDU*	Education (formal education = 1)	8,131	0.691	0.462
*PH*	Mortality rates (represent Public Health)	8,131	0.006	0.002

*IS, IUL, IUZ, IW, IPGDP, IPD, IHB* represents the city indicator where individual located, for example *ISC* indicates whether the individual located in sponge city.

## 4 Empirical analysis

### 4.1 Benchmark regression results

We empirically test the sponge city construction’s direct impact on the urban environment through a benchmark regression based on Equation 1; Table 4 presents the results. In Columns (1) and (2), the regression results are significant at the 1% level, demonstrating that sponge city construction significantly affects the urban environment. In Columns (1), the SC estimated coefficient is 0.109, this means that the construction of sponge cities significantly increased the area of parks or green area in pilot cities by 10.9%. The quality of the built environment has significantly improved. In Column (2), the coefficient of *SC* is negative, indicating that the sponge city pilot policy remarkably decreased the annual average concentration of PM2.5 in pilot cities. After fully considering other factors, the policy reduced the pilot city’s concentration of PM2.5 by 8.98%; The sponge city pilot policy significantly reduced ecological and environmental pollution. Our regression results provide a fundamental basis for further research on the sponge city pilot’s health effects.

**Table 4:** Benchmark regression results for the urban environment.

Variable	(1)	(2)
	PARK	lnPM2.5
*SC*	0.109^***^	−0.0898^***^
	(0.0408)	(0.0321)
Control variables	Y	Y
City	Y	Y
Year	Y	Y
*N*	1,071	1,071
Adjusted *R*^2^	0.975	0.901

*p < 0.1, **p < 0.05, and ***p < 0.01. Cluster-level robust standard errors are in parentheses. Individual fixed effects, year fixed effects, and control variables are considered. These are the same in subsequent tables.

According to Equation 2; Table 5 displays the results from testing the sponge city construction’s effects on individual self-rated health. Column (1) lists the estimation results when control variables are not considered. The estimated coefficient of the core explanatory variable *ISC* is positive and significant at the 1% level. Column (2) presents the estimation results with the control variables; the estimated value of the sponge coefficient is significant and positive. The coefficient in Column (2) is slightly less than that in Column (1), indicating that some of the factors of sponge city construction are absorbed when the control variables are included, and that some factors affect individual health in the control variables. Theoretically, the health effects of sponge city construction can be more accurately estimated by considering time, individual fixed effects, and the control variables. The estimated results reveal that the health levels of individuals located in sponge city pilot have increased by an average of approximately 10.4%.

**Table 5:** Benchmark regression results for the health effects.

Variable	(1)	(2)
	IH	IH
*ISC*	0.129^***^	0.104^**^
	(0.0366)	(0.0407)
Control variables	N	Y
City	Y	Y
Year	Y	Y
*N*	8,131	8,131
Adjusted *R*^2^	0.126	0.155

According to the regression results, in general, sponge city construction in China has significantly improved the urban environment and population health, which supports Hypothesis 1.

### 4.2 Robustness test

#### 4.2.1 Parallel trend tests

The parallel trend assumption is a key prerequisite for estimating the sponge city pilot policies’ effects using a DID model. It was assumed that environmental-level trends should be similar in the pilot and non-pilot cities before the policy’s implementation. To test this assumption, an event study methodology was employed as suggested by Jacobson, LaLonde, and Sullivan (52), with the following econometric model:

(3) $$y_{ct}=\varphi_{0}+\overset{k=4}{\underset{k=-4}{\sum }}\delta_{k}D_{ct}+\lambda_{c}Z_{Ct}+city_{c}+year_{t}+\varepsilon_{ct}$$

In Equation 3 *y* represents the *PARK* or *lnPM2.5*. Here, *D_ct_* contains a series of variables. If the city implemented the sponge city pilot policy in year *t*, the variable took a value of one, and zero otherwise; *k* represents the number of years the pilot policy has been implemented, and *-k* indicates the number of years before implementation. The other variables remain consistent with those of the benchmark model; $\delta_{k}$ represents the regression coefficient in Equation 3, which indicates that the parallel trend assumption holds.

**Figure 2:**
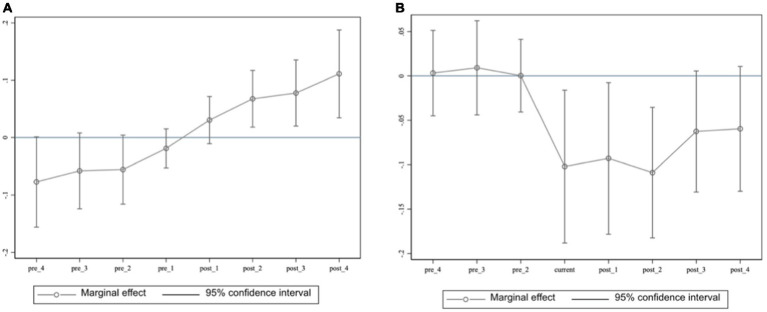
Parallel trend tests. **(A)** *PARK* **(B)** *lnPM2.5.*

Figure 2 shows that for both *PARK* and *lnPM2.5*, all the regression results before the policy implementation are statistically insignificant, suggesting that both groups exhibited similar trends before the sponge city pilot policy’s implementation, with no significant differences. However, the estimated coefficients are significant and non-zero after the implementation. The coefficient for *PARK* increased over the years, while that for the annual average *lnPM2.5* demonstrates an overall decreasing trend. Therefore, the sample successfully passes the parallel trend test.

#### 4.2.2 PSM-DID robustness test

**Figure 3:**
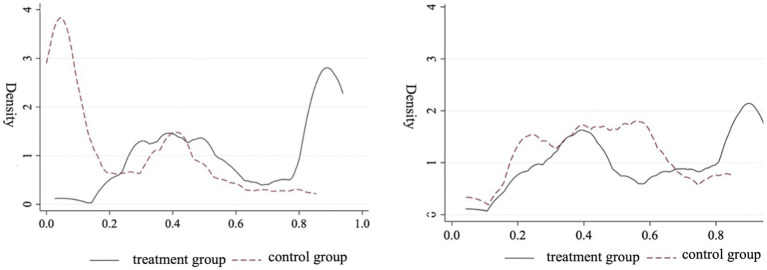
Before-and-after comparison of propensity score distributions.

In China, cities differ significantly, making it challenging to have consistent time effects. Before using the DID method, it is best to select a set of “non-sponge city pilot” controls that closely match the treatment group in case of sample selection bias. This paper used propensity score matching (PSM) to select samples, ensuring that pilot and non-pilot cities for sponge urban initiatives were closely matched in other characteristics to separate the health effects of sponge city pilot policies. The characteristic indicators of *IPGDP*, *IUZ*, *IPD*, and *IHB* were selected for sample matching, a relatively strict intra-caliper (0.005) nearest neighbor matching (1.1) method was employed, and the logit model was applied to estimate each city’s preference score. As Figure 3 illustrates, the probability distributions of the treatment and control groups converged after matching, indicating that PSM method is effective. Based on this, we further adopted the PSM-DID model to evaluate sponge city construction’s impact on individual health.

**Figure 4:**
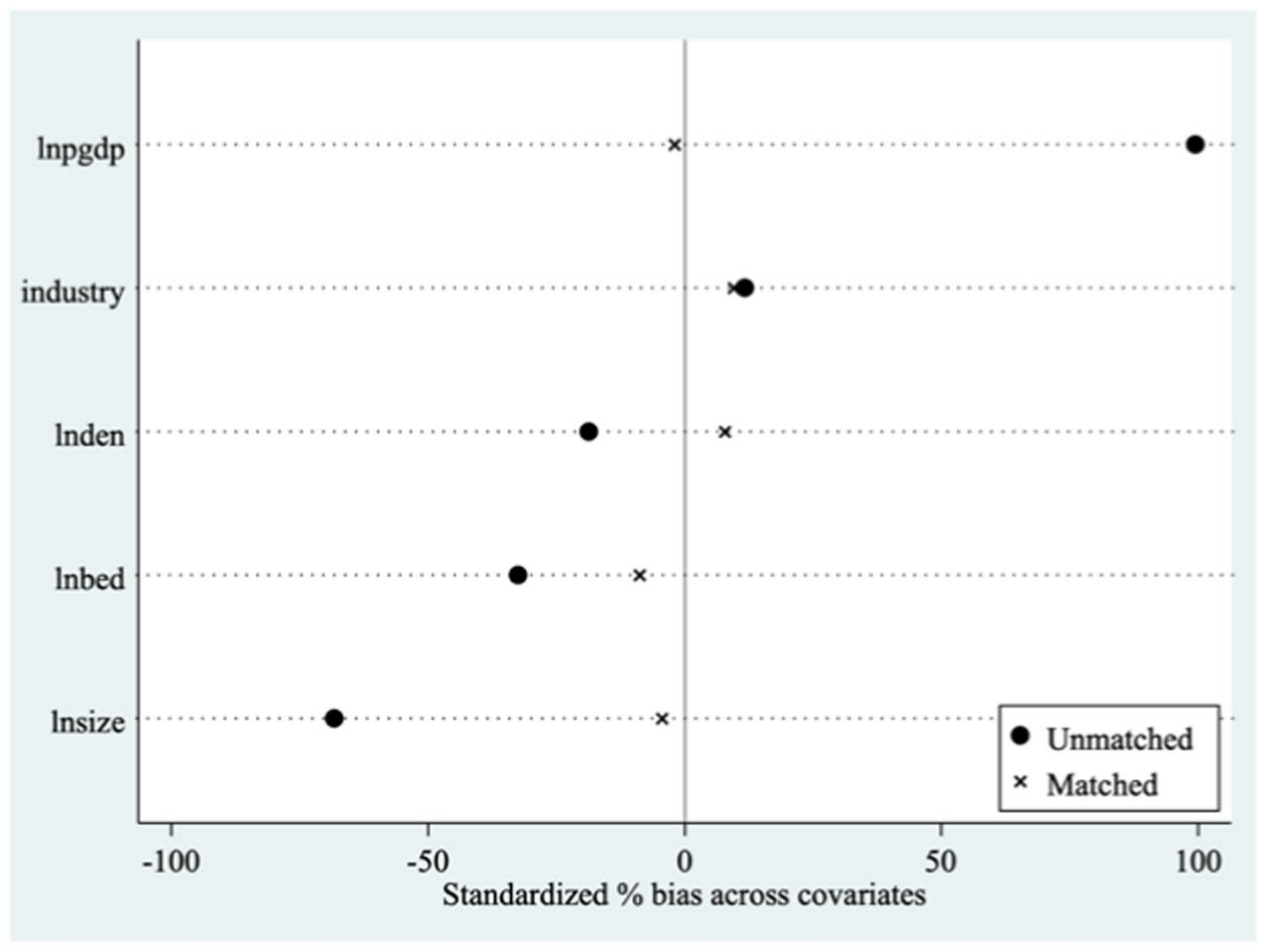
Standardization bias of control variables.

Table 6 presents the results from analyzing the PSM-DID model, which indicate that the construction of sponge cities still significantly promotes population health. After using PSM, the sponge city construction improved population health by 8.75%. The estimation results do not significantly differ from benchmark regression results, further validating the sponge city construction’s health effect in China.

**Table 6:** Robustness test: PSM-DID.

Variable	(1)	(2)
	IH	IH
*ISC*	0.1497^***^	0.0875^*^
	(0.0483)	(0.0511)
Control variables	N	Y
City	Y	Y
Year	Y	Y
*N*	5,589	5,589
Adjusted R^2^	0.0065	0.1062

#### 4.2.3 Sample range test

To ensure the findings’ robustness and mitigate outliers’ impact on the baseline regression results, the samples were regressed using Equation 2 after trimming the top and bottom 5 and 10% of the dependent variable. Table 7 summarizes the results. After removing the outliers, the *ISC* coefficient estimates passed the significance test at the 1% and 5% levels, which is consistent with the baseline estimation results.

**Table 7:** Robustness test.

Variable	(1)	(2)
	IH	IH
	Winsor5%	Winsor10%
ISC	0.111^***^	0.0812^**^
	(0.0391)	(0.0352)
Control variables	Y	Y
City	Y	Y
Year	Y	Y
*N*	8,053	7,441
Adjusted *R*^2^	0.217	0.203

#### 4.2.4 Substitution of explanatory variable

To further support our research conclusion, the mortality rate was substituted for self-rated health to robustness test. The mortality rate (‰) of the city where the individuals resided was used to measure the level of public health (*PH*) during the period from 2011 to 2019. Table 8 demonstrates that after sponge city construction, the mortality rate decreased by 0.985‰, passing the significance test at the 1% level. After adding the control variables, it decreased by 0.632‰. The above results further highlight the sponge city construction’s health effects.

**Table 8:** Robustness test.

Variable	(1)	(2)
	PH	PH
*ISC*	−0.000985^***^	−0.000632^***^
	(0.0001103)	(0.0000739)
Control variables	N	Y
City	Y	Y
Year	Y	Y
*N*	8,131	8,131
Adjusted *R*^2^	0.168	0.495

### 4.3 Analysis of the mechanisms

An empirical analysis revealed that the sponge city pilot construction affects the environment and significantly improves population health. However, the specific underlying mechanisms still require further investigation.

#### 4.3.1 Therapeutic effect

The preceding analysis revealed that sponge city construction can enhance the ecological environment by reducing the concentration of PM 2.5. However, the specific impact mechanisms still need further examination. This paper employs *RW* (Residents ‘wellbeing) to test the therapeutic effect of the ecological environment. The estimated *ISC* coefficient in the first column of Table 9 is 0.0199, it does not pass the significance test. This indicates that, although not statistically significant, there is still a positive correlation between the sponge city construction and residents’ well-being. Expanding on this, when examining the influence of PM2.5 on residents’ well-being, the second column coefficient insignificant. This suggests that while the sponge city construction effectively reduces PM2.5, it does not contribute to a therapeutic effect on individuals’ health. Thus, Hypothesis 1a are not supported.

**Table 9:** Mechanism examination: therapeutic effects.

Variable	(1)	(2)
	SW	SW
*ISC*	0.0199	
	(0.0162)	
Therapeutic effects		0.0403
		(0.0244)
Control variables	Y	Y
City	Y	Y
Year	Y	Y
*N*	6,675	6,675
Adjusted *R*^2^	0.016	0.017

#### 4.3.2 Restorative effect

Benchmark regression results indicate that sponge city construction can enhance the built environment by increasing park or green space area. Numerous studies suggest that urban green spaces have a restorative effect by promoting residents’ outdoor exercise time, contributing to population health. To validate this mechanism, this study selects residents’ average outdoor exercise duration (*OE*) to examine the impact of the restorative effect on individuals’ self-rate health. In Table 10, the coefficient estimate of *ISC* in the first column is 0.0753 and passed the significance test at the 1% level. This suggests that, compared to non-pilot sponge cities, residents in sponge city significantly increase their outdoor exercise time. We further examine the influence of park green space area on residents’ outdoor exercise time. The regression results in column (2) show that the coefficient estimate for park green space area is positive and passed the significance test, confirming that with higher park green space areas, residents tend to have longer outdoor exercise duration. In summary, Hypothesis 1b has been validated.

**Table 10:** Mechanism examination: restorative effect.

Variable	(1)	(2)
	OE	OE
*ISC*	0.0753^***^	
	(0.0168)	
Restorative effect		0.0352^***^
		(0.00796)
Control variables	Y	Y
City	Y	Y
Year	Y	Y
*N*	6,685	6,685
Adjusted *R*^2^	0.016	0.016

## 5 Heterogeneity analysis

### 5.1 Urban characteristic heterogeneity

In addition to the sponge city pilot policy, factors such as the urban administrative level, urbanization level, and geographical location are crucial in determining policy effects. In this subsection, a sample of cities was segmented from different perspectives to explore the sponge cities’ impacts on individual health from a heterogeneous perspective. The city administrative levels were divided by referencing Hang and Cheng (53), whose higher-administrative level cities include sub-provincial cities and provincial capitals; other cities are categorized as lower-administrative level cities. Regarding the urbanization level, we considered the actual context in China to classify cities scored less than 0.6, cities between 0.6 and 0.8, and those scoring 0.8 as having lower-level, medium, and higher-level urbanization, respectively.

#### 5.1.1 Administrative level

Table 11 presents the sponge city pilot policies’ effects on population health in cities with different administrative levels. The results in columns (1) and (2) of Table 11 indicate that the construction of sponge cities has a significant positive impact on individual health in cities with lower administrative levels, while it has no significant effect in cities with higher administrative levels. This suggests that sponge cities construction may have greater benefits in lower-administrative cities, supporting the diminishing marginal effects putting forward in Hypothesis 2.

**Table 11:** Heterogeneity analysis of urban characteristics.

Variable	IH	IH	IH	IH	IH
	higher administrative level	lower administrative level	Higher urbanization level	medium urbanization level	lower urbanization level
	(1)	(2)	(3)	(4)	(5)
*ISC*	−0.0663	0.377^***^	−0.0853	0.312^***^	−0.0781
	(0.0516)	(0.0813)	(0.133)	(0.0674)	(0.127)
*N*	5,338	2,600	2,578	3,411	1,579
Adjusted *R*^2^	0.197	0.314	0.338	0.281	0.380

#### 5.1.2 Urbanization level

Table 11 shows the sponge city policy effect at different levels of urbanization (columns 3–5). The study results indicate that the sponge city construction has no significant impact on the health of residents in cities with either high or low levels of urbanization. However, the sponge city construction has a significant positive impact on cities with medium levels of urbanization, suggesting that the health effect is most evident in rapidly urbanizing cities. Thus, the relationship between sponge cities and individual health is not significant in highly urbanized cities, Hypothesis 3 is not supported.

### 5.2 Regional heterogeneity

China’s regions are unbalanced and vary greatly, and especially between the eastern and central-western regions, with significant differences in resource endowments, economic development, and environmental conditions. Therefore, the heterogeneity of the health effects of sponge city construction requires further study.

This study utilized data from different regions to detect variations in the health effects of the sponge city pilot policies in different regions (Table 12). The *ISC* regression coefficients in Columns (1) and (2) are positive, and the southern cities are significant, while the northern cities are not. Columns (3) to (5) demonstrate that the regression coefficients of the eastern and central cities are positive and pass the significance test, while those of the western region do not. A possible reason for this result is that the precipitation in the southern cities is relatively larger than that in the northern cities, and a sponge city construction significantly contributes to the improvement of local water resources and the ecological environment.

**Table 12:** Heterogeneity analysis of urban characteristics.

Variable	IH	IH	IH	IH	IH
	North	South	East	Central	West
	(1)	(2)	(3)	(4)	(5)
*ISC*	0.0373	0.188^***^	0.195^***^	0.175^*^	−0.168
	(0.0818)	(0.0509)	(0.0611)	(0.105)	(0.110)
*N*	3,563	4,432	3,835	2,151	1,579
Adjusted *R*^2^	0.296	0.257	0.266	0.343	0.456

### 5.3 Analysis of population heterogeneity

In addition to urban-level factors, individual-level factors such as income and household registration (*hukou*) are more important in influencing individual health (54). This study categorizes individuals aged 60 and older into the “older adult” population group, and those under age 60 were analyzed separately. Moreover, we investigated the health effects on local and non-local residents.

#### 5.3.1 Income

The results in Columns (1) and (2) in Table 13 indicate that although the ISC regression coefficient is positive, the sponge city construction’s impact on the health of the older adult population is not significant. However, the impact on the health of those under age 60 passed the significance test at the 5% level. Overall, the construction of sponge city led to an 8.5% improvement in individual health among people under 60 years old.

**Table 13:** Heterogeneity analysis of population characteristics.

Variable	*IH*	*IH*	*IH*	*IH*
	age >60	age ≤ 60	local	non-local
	(1)	(2)	(3)	(4)
*ISC*	0.149	0.085**	0.135**	0.072
	(0.230)	(0.0427)	(0.0543)	(0.0756)
*N*	450	7,132	5,511	2,414
Adjusted *R*^2^	0.449	0.168	0.205	0.338

#### 5.3.2 Hukou status

The results in columns (3) and (4) of Table 13 demonstrate that the construction of sponge cities does not have a significant impact on the individual health of non-local populations, but it significantly improves the health of local people. The construction of sponge cities has increased the individual health level of local residents by 13.5%.

## 6 Discussion

Public policy, especially policy in urban area be proved to have the direct driving force in shaping the urban environment, and thus affect population health (29–32, 55, 56). On the basis of existing research, this study attempts to consider the dual changes of sponge city pilot policy on the ecological environment and the built environment and their impact on population health. This is a topic that few previous studies have explored. Our study not only reveals the influence of sponge city construction on the urban environment but also effectively expands the sponge city pilot policy effect at the micro level. Additionally, this study innovatively combines panel data from 119 prefecture-level cities in China with data from the China Labor-force Dynamics Survey (CLDS), employing a creative approach that integrates micro and macro-level data for empirical research.

Nevertheless, this study’s limitation should be acknowledged. The findings are derived from 119 prefecture-level cities in China from 2011 to 2019. Due to data availability and the timeline of China’s implementation of the sponge city pilot policy, the conclusions are not necessarily universal. For example, although current empirical results indicate that the restorative effects of the built environment significantly promote residents’ health, with the continuous implementation of the sponge city pilot policy, the therapeutic effects of the ecological environment in sponge city construction may play a substantial role on population health over a longer period of time. This aspect needs further exploration in future research.

## 7 Conclusion and policy implications

This is the first study to juxtapose the urban and individual dimensions of sponge city construction effects in contemporary Chinese cities. Linking 2011 to 2019 prefecture-level Chinese city panel data to micro-survey data from the CLDS (2012–2018), we systematically estimated sponge city construction’s health effect; Following a theoretical analysis, this study explored the sponge city pilot policy effects at the urban and individual levels. Therefore, our study consisted of two phases. First, the impact of sponge city pilot policies on the urban environment; Secondly, on this basis, we further study how sponge cities affect population health through the urban environment. The result revealed that: (1) Sponge city construction in China significantly promoted population health, led to an average increase of approximately 10.4%, and PSM-DID robustness tests indicated a significant health effect. (2) The mechanism analysis show that the sponge city construction could promote population health through the restorative effect of the built environment compared to the therapeutic effect of the ecological environment. Although the construction of sponge cities has improved the urban environment, the reduction of PM2.5 has not significantly improved the residents’ wellbeing. In contrast, the increased green park areas did significantly enhance residents’ outdoor exercise time, having a substantial impact on individuals’ health. (3) Heterogeneity analysis shows that, according to the law of “diminishing marginal effect,” the health effect created by the construction of sponge cities reveal that individuals at lower administrative levels have more significant health effects than those in cities with higher administrative levels. As to the individual level, the health effects are significant among the non-older adult population and local residents.

Based on the above conclusions, this paper puts forward several policy recommendations. First, the construction of sponge cities has a positive impact on population health while improving the urban environment. Therefore, it is important to prioritize the health effects of sponge city construction and use them as a measure to promote the healthy cities conception. Second, the health effects of sponge city construction are not benefit by all people, some populations have not benefit from these initiatives. Therefore, sponge city construction must consider population variations. Third, according to the law of diminishing marginal effects, policy planning should focus on addressing the needs of underserved or less-benefited groups, and efforts should be made to improve policy effectiveness and ensure coverage across multiple scales and population categories.

## Data availability statement

Publicly available datasets were analyzed in this study. This data can be found at: CLDS.

## Author contributions

YL: Conceptualization, Methodology, Formal analysis, and Writing the manuscript.
